# Professor Fergus

**Published:** 1838-07

**Authors:** 


### PROFESSOR FERGUS.

On the 3d April, in London, in the twenty-eighth year of his age. Dr. Fergus was professor of forensic medicine in King's College, London; and, although only recently elected to this chair, he showed himself possessed of talents that would have soon raised him to eminence as a teacher. He was greatly respected in private life. Dr. Fergus was born in Fife, and was the son of a minister of the church of Scotland.

